# Does Frailty Predict Health Care Utilization in Community-Living Older Romanians?

**DOI:** 10.1155/2016/6851768

**Published:** 2016-07-17

**Authors:** Marinela Olaroiu, Minerva Ghinescu, Viorica Naumov, Ileana Brinza, Wim van den Heuvel

**Affiliations:** ^1^Foundation Research and Advice on Elderly, Heggerweg 2a, 6176 RB Spaubeek, Netherlands; ^2^Department of Family Medicine, University Titu Maiorescu, Street Pictor Petraşcu 67A, Sector 3, Bucharest, Romania; ^3^Office General Practitioner, Boulevard Dorobantilor, No. 15, Bloc A14, Braila, Romania; ^4^College of Physicians, Street Scolilor, No. 42, Bloc BPP, Sector 5, Braila, Romania; ^5^Research School SHARE, University of Groningen, Heggerweg 2a, 6176 RB Spaubeek, Netherlands

## Abstract

*Background*. The predictive value of frailty assessment is still debated. We analyzed the predictive value of frailty of independent living elderly. The outcomes variables were visits to the general practitioner, hospital admission, and occurrence of new health problems.* Methods*. A one-year follow-up study was executed among 215 community-living old Romanians. General practitioners reported the outcome variables of patients, whose frailty was assessed one year before, using the Groningen Frailty Indicator. The predictive validity is analyzed by descriptive and regression analysis.* Results*. Three-quarters of all participants visited their general practitioner three times more last year and one-third were at least once admitted to a hospital. Patients who scored frail one year before were more often admitted to a hospital. Visits to the general practitioner and occurrence of new health problems were not statistically significant related to frailty scores. The frailty items polypharmacy, social support, and activities in daily living were associated with adverse outcomes.* Conclusions*. The predictive value of frailty instruments as the Groningen Frailty Indicator is still limited. More research is needed to predict health outcomes, health care utilization, and quality of life of frailty self-assessment instruments. Validation research on frailty in different “environments” is recommended to answer the question to what extent contextual characteristics influence the predictive value.

## 1. Introduction

Frailty is considered as common in old age [1]. Frailty indicates a loss of resources on physical, cognitive, and social domains, but a uniform definition does not exist [2]. Frailty is associated with a higher risk for dependence, falls, decreasing quality of life, utilization of care services, depressive symptoms, and mortality in frail old [3–8]. In clinical, inpatient studies frailty indexes are related to various adverse health outcomes [3, 8, 9]. In outpatient adults with cardiovascular diseases frailty is a predictor for disability and mortality [10]. In primary health care screening on frailty may discover unrecognized health problems [11], and geriatric intervention based on identified frail old through screening in primary health care may reduce risks of hospitalization [4, 5, 11]. Kiely et al. showed that frailty in community-living elderly had a predictive value for the incidence of recurrent falls, overnight hospitalization, emergency room visit, and the prevalence of disability, chronic diseases, self-reported health, and cognitive functioning [12].

Frailty is very common in older people. The overall weighted prevalence of frailty in community-dwelling old citizens is 10.7%, but it varies between countries from 4.0% to 59.1% [13, 14]. In Europe, the highest mean frailty index scores are found in Italy, Spain, and Poland and the lowest in Denmark, Switzerland, and Ireland [14]. The prevalence of frailty is strongly related to national economic indicators, showing that the prevalence of frailty is lower in higher-income countries [15].

Screening on frailty is important [11, 12]. An overview study identified 11 instruments with the potential to be used to assess frailty in primary health care settings [2]. Only six of these were validated. However, the validity of the same instrument varies between different studies, which may be related to the study design. Although they all are based on community-dwelling elderly, the age, context, and country/region do vary [8, 11, 12, 16, 17]. Despite the variety in assessment instruments, consensus is growing that simply and easily executing screening on frailty might allow physicians to objectively recognize frail persons [18]. The predictive value of these instruments is still a matter of debate. The value of frailty to predict adverse health outcomes is defined as a major research question in the European Union, related to the aging of its population [19].

Our research is executed in Romania, which is interesting for various reasons, while most studies on predictive validity are executed in well-structured health care systems [2, 4, 17, 19–22]. The context of the health care system and the role of the GP may affect the predictive value of frailty assessment instruments. The Romanian context differs considerably in infrastructural facilities, health status of old people, and health care arrangements as compared to the studies published so far [22].

The Romanian population is one of the fastest aging populations in Europe, whereas the total population is simultaneously decreasing. This number will increase by 5.4% in the coming decade, while the total population will decrease by 3.1% [22]. Long-term care facilities are lacking, which would necessitate interventions to prevent dependency. During the last decade, health policy has been directed to strengthening the role of primary health care, to reducing (unnecessary) hospitalization, and to encouraging preventive screening programs [22]. Simultaneously, the Romanian elderly population has been confronted with dramatic changes in social security (reductions in pension plans and in free access to health care) as well as in the quality of health care (waiting lists, a lack of personnel and facilities, copayments). Research showed that such measures have affected negatively the health status of old Romanians [22]. Because personal and environmental conditions may affect frailty self-assessment, considering these factors is needed also in studying the predictive value of self-assessed frailty instruments [23].

## 2. Material and Methods

### 2.1. Design and Sampling

This study used a prospective design to analyze the predictive value of frailty in primary health care. We used, as outcomes, the number of general practitioner (GP) visits and hospital admissions and the occurrence of new health problems. These outcomes are easy to register and reliable because they are part of the GPs registration system.

This study is executed in the Braila district, situated in eastern Romania, which has 230.000 inhabitants. All 145 general practitioners (GPs) received an invitation letter by the Regional College of Physicians to participate in a study to assess frailty in community-living old Romanians [24]. Twenty-two GPs expressed their willingness and did participate in the research. They were asked to send a frailty self-assessment questionnaire, the Groningen Frailty Indicator (GFI), to 10 randomly selected patients of 65 years and over in their practice. The 10 patients were selected from the patients list of all patients of 65 years and over by randomly selecting one patient and then every tenth till 10. The letter asked for informed consent and requested to complete and return the questionnaire. To assess frailty the Groningen Frailty Indicator (GFI) was used. In total 215 questionnaires were returned.

One year after frailty was assessed, the GPs were asked for information on the health care utilization of the participating patients. Three indicators were used for health care utilization: did the patient consult the GP in the year after the frailty was assessed (and if so how often), was the patient admitted to a hospital in the year after the frailty was assessed (and if so how often), and/or did the patient develop a new health problem in the year after the frailty was assessed (no/yes and if yes what kind of problem) and if so what kind of problem. All but two GPs send back this information, so 20 patients were lost in follow-up. The data of five patients were incomplete and therefore had to be excluded for further analysis. In total we included 190 patients. No statistically significant differences were found between the sociodemographic characteristics and frailty scores of the 25 excluded patients as compared to the 190 patients.

### 2.2. Measures

The GFI has been identified as a well-validated instrument to assess frailty and tested in multiple settings [2, 4, 18, 20, 24, 25]. It is a 15-item screening instrument assessing four domains of functioning and resources: physical, cognitive, social, and psychological [2, 5]. The GFI is a 15-item self-assessment screening instrument assessing functioning and resources in the domains: physical (9 items, e.g., shopping, dressing, and toileting), cognitive (1 item, i.e., memory), social (3 items, e.g., network and getting attention), and psychological (2 items, i.e., feeling sad and being calm). The items and answer categories are variably positive or negative formulated, and the answer categories vary from yes/no to a 0–10 scale (for details see [20]). The designers of the GFI recommend dichotomizing the answer categories by 0-1, with 1 indicating a dependency problem. The total score may vary between 0 and 15, and a score of 4 or higher is considered to indicate “moderate” or “severe” frailty by the designers of the GFI [20]. A validation study of the GFI in Romania recommended to use a score >5 as indication for frailty [24]. In addition to the GFI we asked participants for age (in years), gender (female-male), marital status (married, divorced, widowed, never married, and other), and whether they lived in an urban or rural area.

### 2.3. Data Analysis

We used SPSS-20 to analyze the data. We presented the frequencies of sociodemographic data (in categories) and frailty scores according to the GFI. As dependent variables we used frequency of visits to the GP in the last year (number of visits, scores 1 to 4), frequency of hospital admission in the last year (number of admissions, scores 1 to 3), and occurrence of new health problem in the last year (scores 1 to 3 and type of problems) (Table 1).

First we described the sociodemographic variables, frailty score, and outcome variables and the statistically significant bivariate relationships. We analyzed the relationship between sociodemographic data and GFI frailty scores and the three dependent variables by stepwise linear regression analysis. Regression model solutions were checked for collinearity. Also we explored the role of individual items of the GFI in outcome variables through bivariate analysis and report the statistically significant correlations.

## 3. Results

The sociodemographic variables showed a representative picture of the older Romanians in the region, with the exception of urban living: older Romanians living in the city were overrepresented in the sample. Frailty scores were relatively high: 48% was assessed as frail, following the recommended cut-off point of a frailty score of 6 points or more.

Older Romanians visited their GP frequently, that is, three-quarters went for consultation or control three times of more during the last year, while 9.5% did not visit their GP during the last year.

One-third of the participants were at least once admitted to a hospital during the last year and three out of ten older Romanians developed a new health problem during the last year. The following health problems were most frequently mentioned: psychogeriatric problems (4.3%), heart diseases (3.1%), and stroke (2.5%).

Older old citizens scored higher on the GFI than younger ones. However, older citizens did not visit their GP more frequently as compared to younger ones; on the contrary: young-old patients did visit their GP more frequently as old-old patients (Pearson's *r* = −0.194, *p* < 0.01).

GFI frailty scores correlated statistically significant to frequency of hospital admission (Pearson's *r* = 0.163, *p* < 0.05), but not to GP visits or the occurrence of new health problems in the last year. Old Romanians, who were admitted to the hospital in the last year, had been more frequently scored as frail by the GFI one year before.

Hospital admission was strongly related to the occurrence of new health problems (Pearson's *r* = 0.367, *p* < 0.01). The occurrence of new health problems was also statistically significant related to visits to the GP (Pearson's *r* = 0.233, *p* < 0.01).

Multivariate analysis showed low predictive value of sociodemographic data on GP visits, hospital admission, and new morbidity with the exception of age, which was significantly related to GP visits.

The GFI forecasted hospital admission also when sociodemographic data were taken into account (see Table 2). Persons with high GFI scores, that is, frail one year ago, were more frequently admitted to the hospital in the last year.

Visits to the GP and occurrence of new health problems in the last year were not statistically significant related to GFI frailty scores one year before in the regression analysis.

At item level we looked for statistically significant associations between each GFI item and health care utilization. Various statistically significant associations were found. The use of more medicines (polypharmacy as assessed by the GFI) was strongly related to both more GP visits and more hospital admissions (Pearson's *r* = −0.275, *p* < 0.01, and *r* = −0.232, *p* < 0.01, resp.).

Receiving social support was significantly related to GP visits, hospital admission, and the occurrence of new health problems (Pearson's *r* = 0.168, *p* < 0.05, *r* = −0.174, *p* < 0.05, and *r* = −0.152, *p* < 0.05, resp.): well supported old citizens did visit the GP more frequently, while less supported old citizens were admitted more frequently to the hospital and developed a new health problem during the last year.

Older Romanians without problems in activities of daily living did visit their GP more frequently, while those with problems in daily activities were admitted more frequently to the hospital and developed more frequently new health problems (Pearson's *r* = −0.195, *p* < 0.01, *r* = −0.148, *p* < 0.05, and *r* = −0.172, *p* < 0.05, resp.).

## 4. Discussion

The findings of this prospective study indicated a predictive value of the GFI for hospital admission, which is confirmed in other studies [4, 20]. The occurrence of new health problems had also an important effect on care utilization. In the literature it is recommended to take into account (new) morbidity in explaining care utilization [26]. Indeed, when we additionally analyzed the bivariate correlation (Pearson's *r* = 0.287, *p* < 0.01  and  *n* = 124) between GFI scores and hospital admission for elderly who had developed a new health care problem the predictive value was stronger.

The* absence* of a significant relationship between frailty and GP visits in our study was remarkable as was the direction of the relationship. Most studies found a positive association between frailty scores and GP visits [4, 20]. However, our study showed that* less* frail elderly did visit their GP more frequently. Also we found that old Romanians without problems in activities of daily living did visit their GP more frequently. This could be explained by difficulties frail patients experience to reach their GP for consultation, which may be specific to the Romanian context, due to absence of appointment consultation, transportation difficulties, and copayment. This underlines the importance of taking into account the role of contextual characteristics (infrastructure, social support and security systems, and economic development) in the predictive value of frailty scores.

Thirty percent of older Romanians developed a new health problem during the last year, but no significant association was found with the frailty score one year before. This could be explained in relation to the finding that more frail old Romanians visited their GP less frequently, because they could not visit their GP by themselves. The GP might not be aware of new health problems in these patients.

Various items of the GFI had a significant correlation with health care utilization. The relationship between more medicines and both more GP visits and more hospital admissions indicates the risks of polypharmacy and underlines the importance of polypharmacy control. Maybe the frequent visit to the GP of polypharmacy patients may be seen as a control visit to check for side effects of polypharmacy. Although patients with polypharmacy often have multiple comorbidities, which affects their risk on health complications, polypharmacy itself is also related to various health complications as weight loss and reduced walking speed [27].

Interesting is the finding that old citizens with sufficient social support did visit their GP more frequently, while less supported old citizens were admitted more frequently to the hospital and had developed new health problems. Could it be that old citizens with sufficient support are “advised” to visit their GP “in time,” while less supported elderly stayed at home till they were admitted to the hospital? Other studies showed that GP visits by patients were significantly and positively related to* activities of daily living* (ADL) problems [4, 12]. However, this study showed that in the Romanian context community-living elderly with* no* ADL problems did visit their GP more frequently. This finding is in line with what we mentioned before; that is, less frail old did visit their GP more often. Is it because they still were mobile and active and/or were stimulated by their partner or “social environment” to see their doctor? And on the other hand, we have to realize that Romanian patients are rarely visited at home by the GO patients at home.

The findings about the relationship between individual GFI items and outcome variables indicated that looking at these items may be worthwhile, especially in clinical practice, which raises the question whether individual items in frailty scales should be weighted to assess an overall frailty score [12, 28, 29].

As all studies this one had some strong and weak points. Strong points included the longitudinal design and the Romanian context. Validity studies were executed until now in health care systems and cultural context which were alike. As explained in Section 1, the Romanian context differed in various ways, which indicates that health care system and cultural context affect the prevalence of frailty as well as its predictive value [14, 22]. Another strong point is the willingness of patients to participate in health studies as is found in various studies in central-eastern European countries. A point of consideration is the representativeness of the sample because the sample was taken indirectly (through GPs patient lists) and from one district. We do not believe that the Braila district differs significantly from the other Romanian districts, but still we would recommend a national study on frailty, sampled on population base (65 years and over). The more because the number of elderly living in rural areas maybe underrepresented in our study. In rural areas less GPs are available.

A weak point of the study was that we could not assess changes in frailty or disability during the last year. Changes in frailty scores in the last year could be related to higher health care utilization. Another aspect to be mentioned is the loss of 25 patients in our follow-up. However, as we mentioned, no significant differences were found between the characteristics of the 25 and 190 participants, respectively.

## 5. Conclusions

We conclude that the predictive value of the GFI in primary health care is limited, which is in line with other findings [1, 2, 4, 25]. More research is needed to predict health outcomes, health care utilization, and quality of life of frailty self-assessment instruments in primary health care [19].

We recommend to execute validation research on frailty in different “environments” to answer the question to what extent contextual characteristics influence the predictive value of frailty. Validated and simple self-assessment screening tests allow health care workers to recognize frail elderly [18]. In primary health care such recognition opens possibilities for (preventive) actions as well as the identification of treatable health problems [16, 25, 30].

## Figures and Tables

**Table 1:** Frequencies of sociodemographic data, visits to the general practitioner (GP), hospital admission, and occurrence of new health problems by frailty scores on Groningen Frailty Indicator (GFI), in %; *n* = 190.

Variable	Score	Frailty score GFI		Percentages (total 100%)
		Not frail	Frail	
Age	65–69 years	23.5%	20.6%	22.1%
	70–74 years	31.6%	26.1%	29.0%
	75–79 years	30.6%	28.3%	29.4%
	80 years or >	14.3%	25.0%	19.5%

Gender	Women	60.2%	70.7%	65.3%
	Men	39.8%	29.3%	34.7%

Marital status	Married	60.2%	55.4%	57.9%
	Widowed	39.8%	43.4%	41.6%
	Not married	0.0%	1.2%	0.5%

Urban-rural living	Urban	78.6%	72.5%	75.3%
	Rural	21.4%	27.5%	24.7%

Frequency of visits to GP in the last 12 month	No visit	7.1%	12.1%	9.5%
	1 visit	6.1%	6.5%	6.3%
	2 visits	10.2%	9.7%	10.0%
	>2 visits	76.5%	71.7%	74.2%

Hospital admission in the last 12 months	None	73.5%	60.9%	67.4%
	1 admission	19.4%	31.5%	25.3%
	2 admissions+	7.1%	7.6%	7.4%

Occurrence of a new health care problem	No	67.3%	63.1%	65.2%
	Do not know	9.2%	9.5%	7.9%
	Yes	23.5%	30.4%	26.9%

**Table 2:** Final models of stepwise linear regression analysis with frailty scores by Groningen Frailty Indicator (GFI) as predictive variable and visits to the general practitioner (GP) and hospital admission over the last year as outcome variables (standardized coefficients beta are presented).

Variables	GP visits	Hospital admission	New health problem
Age	−0.175^*∗*^	0.002	0.149
Gender	0.096	0.074	−0.022
Marital status	−0.076	0.053	−0.114
Urban/rural living	−0.019	0.031	−0.145

GFI score	−0.076	0.146^*∗*^	0.107

Adjusted *R* squared	0.043	0.002	0.027

^*∗*^Statistically significant at < 0.05 level.
